# From TNM 8 to TNM 9: Stage Migration and Histology-Specific Patterns in Lung Cancer

**DOI:** 10.3390/cancers17203290

**Published:** 2025-10-10

**Authors:** Amalia Constantinescu, Radu-Nicolae Căprariu, Emil-Robert Stoicescu, Roxana Iacob, Marius Mânzatu, Janet Camelia Drimus, Alessia-Stephania Roșian, Alexandre Ionescu, Cristian Oancea, Diana Manolescu

**Affiliations:** 1 Doctoral School, “Victor Babeș” University of Medicine and Pharmacy Timisoara, Eftimie Murgu Square No. 2, 300041 Timisoara, Romania; amalia.constantinescu@umft.ro (A.C.); janet.drimus@umft.ro (J.C.D.); alessia.rosian@student.umft.ro (A.-S.R.); alexandre.ionescu@outlook.com (A.I.); 2 Department of Radiology and Medical Imaging, “Victor Babeș” University of Medicine and Pharmacy Timisoara, Eftimie Murgu Square No. 2, 300041 Timisoara, Romania; dmanolescu@umft.ro; 3 Research Center for Medical Communication, “Victor Babeș” University of Medicine and Pharmacy Timisoara, Eftimie Murgu Square No. 2, 300041 Timisoara, Romania; roxana.iacob@umft.ro; 4 Research Center for Pharmaco-Toxicological Evaluations, “Victor Babeș” University of Medicine and Pharmacy Timisoara, Eftimie Murgu Square No. 2, 300041 Timisoara, Romania; 5 Field of Applied Engineering Sciences, Specialization Statistical Methods and Techniques in Health and Clinical Research, Faculty of Mechanics, “Politehnica” University Timisoara, Mihai Viteazul Boulevard No. 1, 300222 Timisoara, Romania; 6 Department of Anatomy and Embryology, “Victor Babeș” University of Medicine and Pharmacy Timisoara, Eftimie Murgu Square No. 2, 300041 Timisoara, Romania; 7 Clinical Hospital of Infectious Diseases and Pneumophthisiology “Dr. Victor Babeș” Timișoara, Gheorghe Adam No. 13, 300226 Timisoara, Romania; marius_manzatu@yahoo.com; 8 Center for Research and Innovation in Precision Medicine of Respiratory Diseases (CRIPMRD), “Victor Babeș” University of Medicine and Pharmacy Timisoara, Eftimie Murgu Square No. 2, 300041 Timisoara, Romania; oancea@umft.ro; 9 Department of Pulmonology, “Victor Babeș” University of Medicine and Pharmacy Timisoara, Eftimie Murgu Square No. 2, 300041 Timisoara, Romania

**Keywords:** lung cancer, TNM classification, stage migration, histology, adenocarcinoma, N2 subdivision

## Abstract

Introduction: The 9th edition of the TNM classification for lung cancer implemented significant revisions, notably the subdivision of the N2 and M1c categories, to enhance anatomical precision and prognostic accuracy. Nonetheless, the actual effects of these modifications on stage distribution, histology-specific patterns, and clinical interpretation remain to be fully evaluated. Objectives: To compare lung cancer staging distributions between the 8th and 9th TNM editions, analyze patterns of stage migration, and evaluate histology-specific reclassification trends. Although TNM 9 applies the same descriptors across all histological subtypes, the magnitude of stage migration varies. In our cohort and in international datasets, adenocarcinoma demonstrated a higher likelihood of reclassification into advanced stages compared to other subtypes. Methods: A retrospective analysis was performed on a cohort of lung cancer patients staged according to the 8th and 9th editions of the TNM classification. Stage distribution alterations were analyzed by chi-squared tests, whereas McNemar’s test examined the directional shifts in upstaging and downstaging. Further investigations evaluated the correlation between histological subtype and stage reclassification. Results: A statistically significant redistribution of stages was noted (χ^2^ = 1013.03, df = 64, *p* < 0.0001), with a notable prevalence of upstaging (*p* = 0.0019). The most significant proportional increase was observed in stage IIIA, mostly attributable to the N2 subdivision (N2a vs. N2b). Adenocarcinoma was the predominant histological subtype at all stages and showed a greater tendency for reclassification into advanced stages, specifically IIIA and IIIB. Squamous cell carcinoma was predominantly observed in stages IIB and IIIA, whereas small cell and large cell carcinomas were concentrated in advanced stages. These histology-specific patterns correspond with international findings, including research confirming the prognostic relevance of N2 subdivision. Conclusions: The 9th edition of the TNM classification results in significant stage migration, particularly in adenocarcinoma cases, indicating the improved sensitivity of the updated criteria in identifying advanced nodal disease. These modifications significantly impact prognostic evaluation and global comparability of clinical cohorts, supporting the implementation of TNM 9 as a more anatomically and biologically relevant staging system.

## Simple Summary

The TNM classification is an essential instrument for assessing the stage of lung cancer, informing prognosis and therapy strategies. In 2024, the 9th edition implemented significant modifications, notably the division of the N2 category into single-station (N2a) and multi-station (N2b) nodal illness, as well as the clarification of the concept of distant metastases (M1c1 vs. M1c2). Specifically, M1c1 refers to multiple metastatic deposits confined to a single extrathoracic organ, whereas M1c2 designates multiple metastatic deposits involving different extrathoracic organs. Our study examined lung cancer staging according to the 8th and 9th editions. The revised system resulted in considerable stage migration, with an increased number of patients categorized into advanced stages, particularly stage IIIA. Adenocarcinoma was the predominant histological type and showed an increased risk for progression to later stages relative to other subtypes. These findings correspond with international research and support the 9th edition as a more accurate and biologically relevant staging system, enhancing the consistency of prognostic evaluation and clinical decision-making.

## 1. Introduction

### 1.1. Worldwide Prevalence of Lung Cancer

Lung cancer constitutes a significant public health challenge and persists as the primary cause of cancer-related mortality globally. GLOBOCAN 2020 reports around 2.2 million new cases and approximately 1.8 million deaths each year, constituting 18% of all cancer deaths. With an overall 5-year survival of about 18% worldwide, survival rates are still quite low despite advancements in prevention, screening, and treatment [1,2].

Non-small cell lung cancer (NSCLC) accounts for approximately 85% of all lung malignancies, with adenocarcinoma being the most common histological subtype, followed by squamous cell carcinoma and large cell carcinoma. Small cell lung cancer (SCLC) constitutes the remaining 15% and is characterized by a more aggressive progression and early metastatic dissemination [3,4].

Epidemiological patterns differ with geography, influenced by tobacco use, environmental exposures (such as air pollution and occupational carcinogens), and the execution of screening programs. While smoking cessation and advancements in treatment have resulted in small reductions in incidence and death in high-income nations, the burden on low- and middle-income countries is growing [5,6].

Early-stage detection is uncommon, as most patients are diagnosed with advanced disease, therefore limiting curative treatment alternatives. Accurate disease staging is essential for guiding treatment options, assessing prognosis, and establishing patient management across institutions and nations. The Tumor–Node–Metastasis (TNM) classification, regularly revised by the International Association for the Study of Lung Cancer (IASLC), serves as the universally recognized system for lung cancer staging, facilitating precise collaboration among clinicians and facilitating significant comparison of outcomes globally [7].

### 1.2. Key Updates from TNM 8 to TNM 9

Despite significant research advancements and the availability of numerous treatment options, lung malignancies continue to be the leading cause of cancer-related deaths worldwide, irrespective of gender. Therefore, the development of a universal nomenclature used to define the extent of lung cancer has been crucial for establishing individualized treatment plans for each patient [8].

The TNM staging system is a globally acknowledged and periodically revised tool used for providing accurate clinical staging, as well as enhancing the prediction of therapy outcomes. The ninth edition of the TNM classification of lung cancer has been implemented starting January 2025 and is based on evidence collected from a large international database, comprising 124,581 cases gathered from 25 countries [9].

The updates brought by the 9th TNM edition are as follows [10,11]:

T descriptor

The new edition has validated the descriptors introduced by the previous edition that pertain to the characteristics of the primary tumor; hence, no additions were made with regard to the T category.

N descriptor

The N2 category, which implies the presence of metastatic disease affecting the ipsilateral mediastinal and/or subcarinal nodal stations, has been split into

N2a—involvement of a single N2 station;
N2b—involvement of multiple N2 stations. In the 9th TNM edition, it is worth noting that nodal disease is measured by the number of the involved nodal stations, rather than the total number of lymph nodes affected.

M descriptor

The M1c subcategory has been divided into its component parts:

M1c1—multiple metastatic deposits affecting a single extrathoracic organ;
M1c2—multiple metastatic deposits affecting multiple extrathoracic organs.

### 1.3. Role of TNM Classification in Clinical Decision-Making

Stage groups

Taking into account the aforementioned revisions, a more precise stratification of lung cancers has been implemented by reordering the TNM combinations encompassing certain stage groups [10,11]:

- T1N1 tumors: downstaged from IIB to IIA (Figure 1 and Figure 2);
- T1N2a tumors: downstaged from IIIA to IIB (Figure 3 and Figure 4);
- T2N2b tumors: upstaged from IIIA to IIIB (Figure 5, Figure 6, Figure 7 and Figure 8);
- T3N2a tumors: downstaged from IIIB to IIIA (Figure 9 and Figure 10).

Nonetheless, stage IV was not affected by these rearrangements, due to the fact that M1c1 and M1c2 have both been assigned to stage IVB, consistent with the M1 classification of the previous edition.

The downstaging draws special attention to the expanded treatment options available for patients presenting with lung cancer, even in cases that were previously regarded as too advanced for surgical resection. For example, the treatment options for stage IIIA NSCLC include a combination of surgery, chemotherapy, radiation therapy, immunotherapy, as well as targeted therapies. In contrast, stage IIIB NSCLC is frequently deemed unsuitable for surgical intervention, and treatment options typically revolve around chemotherapy and radiation therapy. Therefore, T3N2a NSCLC tumors, having been downstaged from IIIB to IIIA, could now be amenable to surgical intervention, in addition to the previously mentioned non-surgical treatments [12].

## 2. Materials and Methods

### 2.1. Study Design and Patient Selection

We conducted a retrospective study including patients diagnosed with primary lung cancer and staged according to both the 8th and 9th editions of the TNM classification. All cases were managed at Dr. Victor Babeș Hospital of Infectious Diseases and Pneumophtisiology, Timișoara, Romania, between January 2025 and July 2025.

Inclusion criteria were:

- Histologically confirmed primary lung cancer, diagnosed through CT-guided transthoracic lung biopsy;
- Complete clinical, imaging, and staging data available for both TNM editions;
- No prior treatment before staging evaluation.

Exclusion criteria included:

- Incomplete diagnostic work-up;
- Previous history of lung malignancy;
- Presence of other malignancies;
- Synchronous primary tumors.

**Figure 11:**
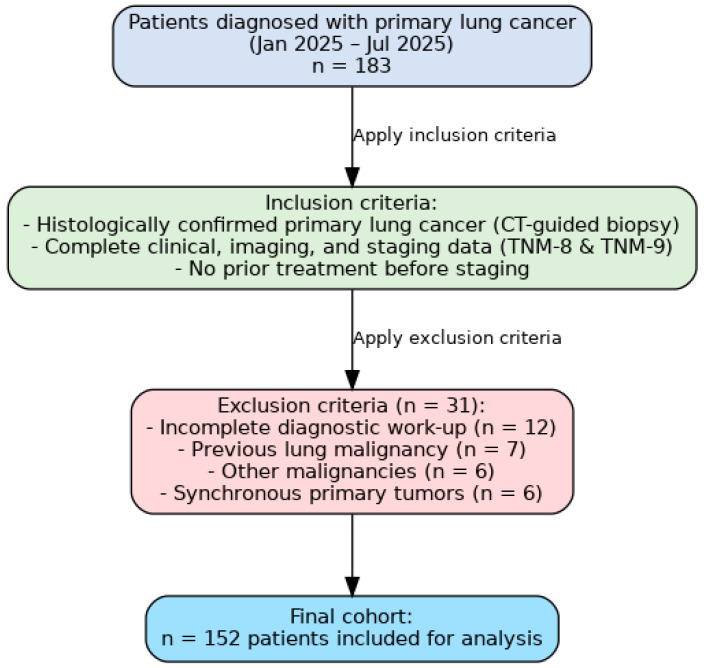
Flowchart of inclusion and exclusion criteria applied to the study population.

After applying these criteria, 152 patients were included in the final analysis (Figure 11).

### 2.2. Data Collection and Imaging Evaluation

For each patient, demographic data, smoking history, comorbidities, histological subtype, and tumor characteristics were extracted from electronic medical records (Table 1).

Radiological staging included contrast-enhanced chest computed tomography (CT) for all patients, supplemented with positron emission tomography-computed tomography (PET-CT) and/or brain magnetic resonance imaging (MRI) when clinically indicated. Mediastinal staging was performed according to institutional protocols and IASLC recommendations. All imaging studies were reviewed independently by two thoracic radiologists, blinded to the histopathological results, to ensure consistency in TNM assignment, using RadiAnt DICOMViewer 2025.2 (Medixant: Poznań, Poland, https://www.radiantviewer.com/). Discrepancies were resolved by consensus.

All CT-guided transthoracic lung biopsies were performed under sterile conditions using coaxial needle systems and TRU-CUT needle biopsy, with histopathological diagnosis established according to the latest WHO Classification of Thoracic Tumours, 5th edition (2021) [13,14].

### 2.3. Staging Procedures

Staging was determined using clinical, radiological, and pathological data following the IASLC guidelines. For each patient, classification was first assigned according to the 8th edition of the TNM staging system, then reassessed according to the updated criteria of the 9th edition.

The main changes incorporated into the reclassification included:

N descriptor: subdivision of N2 disease into single-station (N2a) and multi-station (N2b);
M descriptor: subdivision of M1c into M1c1 (multiple lesions in a single extrathoracic organ) and M1c2 (multiple lesions in multiple extrathoracic organs);
Corresponding updates in stage grouping.

In cases where both imaging-based and pathology-based staging were available, pathological staging prevailed.

### 2.4. Statistical Analysis

The primary outcome was the extent and direction of stage migration between TNM 8 and TNM 9 (upstaging, downstaging, or unchanged stage). A secondary outcome was the distribution of histological subtypes across TNM 9 stages and their association with reclassification patterns.

Stage distribution differences were assessed using MedCalc^®^ Statistical Software version 23.0.9 (MedCalc Software Ltd., Ostend, Belgium) for chi-squared and McNemar tests. Data visualization and figure generation (stacked bar charts, paired bar plots, boxplots, alluvial diagrams) were performed in Python (v3.11), using pandas, matplotlib, seaborn, and networkx libraries. TNM stages were mapped to an ordinal numerical scale for statistical plotting when appropriate, and image outputs were exported as high-resolution PNG files using matplotlib. All analyses were conducted in a Jupyter Notebook 7.4.7 environment, and the full code is available upon request.

### 2.5. Ethical Considerations

The study was conducted in accordance with the principles of the Declaration of Helsinki and approved by the Ethics Committee of ‘Dr. Victor Babeș’ Hospital of Infectious Diseases and Pneumophtisiology, Timișoara, Romania (approval number 4903/30 May 2025). Given the retrospective design and anonymization of data, the requirement for individual informed consent was waived.

## 3. Results

### 3.1. Comprehensive Alterations in Stage Distribution

A comparison of TNM 8 and TNM 9 staging distributions revealed significant changes, with the most notable increase occurring in stage IIIA (from 15 to 23 cases). A chi-squared test confirmed statistical significance (χ^2^ = 1013.03, df = 64, *p* < 0.0001). These findings indicate that the updated descriptors have a measurable impact on patient reallocation across stages.

**Figure 12:**
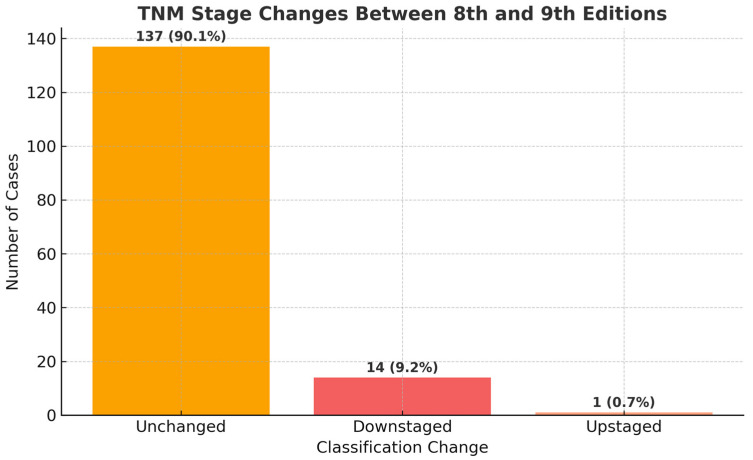
Distribution of TNM stage changes between the 8th and 9th editions. The bar chart illustrates the number of patients whose staging remained unchanged, as well as those who were upstaged or downstaged according to the 9th edition. The distribution indicates a significant predominance of upstaging (*p* = 0.0019, McNemar test).

We employed McNemar’s test to evaluate the direction and extent of reclassification between TNM editions by comparing discordant staging pairs. The analysis indicated a statistically significant difference (χ^2^ = 9.60, *p* = 0.0019), with a higher incidence of cases being downstaged compared to those upstaged in the transition from the 8th to the 9th edition. The corresponding bar chart (Figure 12) illustrates the quantity of unchanged, upstaged, and downstaged cases, highlighting the clinical significance of these staging changes.

### 3.2. Comprehensive Reclassification Patterns

**Figure 13:**
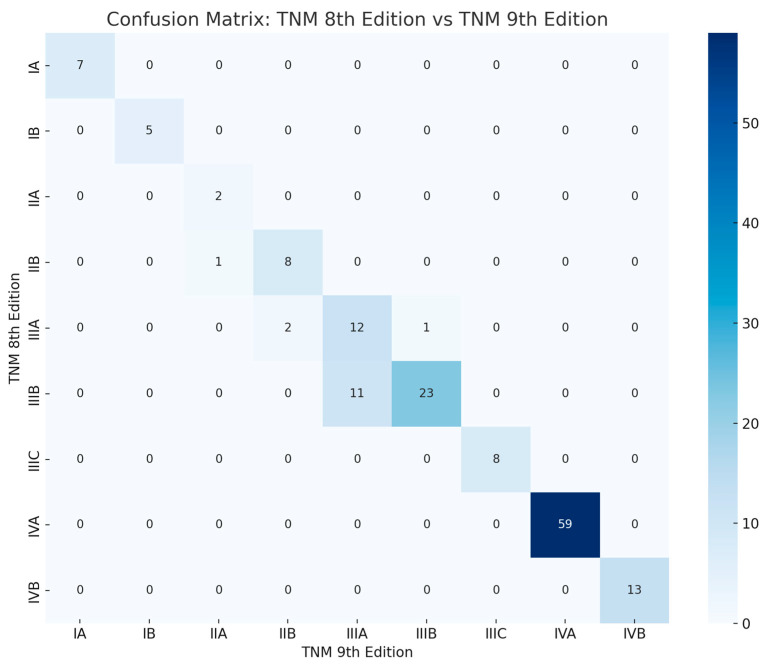
Confusion matrix showing the distribution of TNM stages between the 8th edition (rows) and the 9th edition (columns). Each cell represents the number of patients who were classified under a given stage in the 8th edition and reclassified in the 9th edition. Patients on the diagonal remained in the same stage. Cells above the diagonal represent Up-staged patients (from TNM 8 to TNM 9), while cells below the diagonal represent Down-staged patients.

A confusion matrix (Figure 13) illustrates the distribution of patients between TNM 8 and TNM 9 stages, highlighting both unchanged classifications (diagonal) and reclassifications (off-diagonal). Several cases underwent stage migration, confirming the influence of the updated criteria on reallocation patterns.

### 3.3. Visual Comparison of Stage Proportions

**Figure 14:**
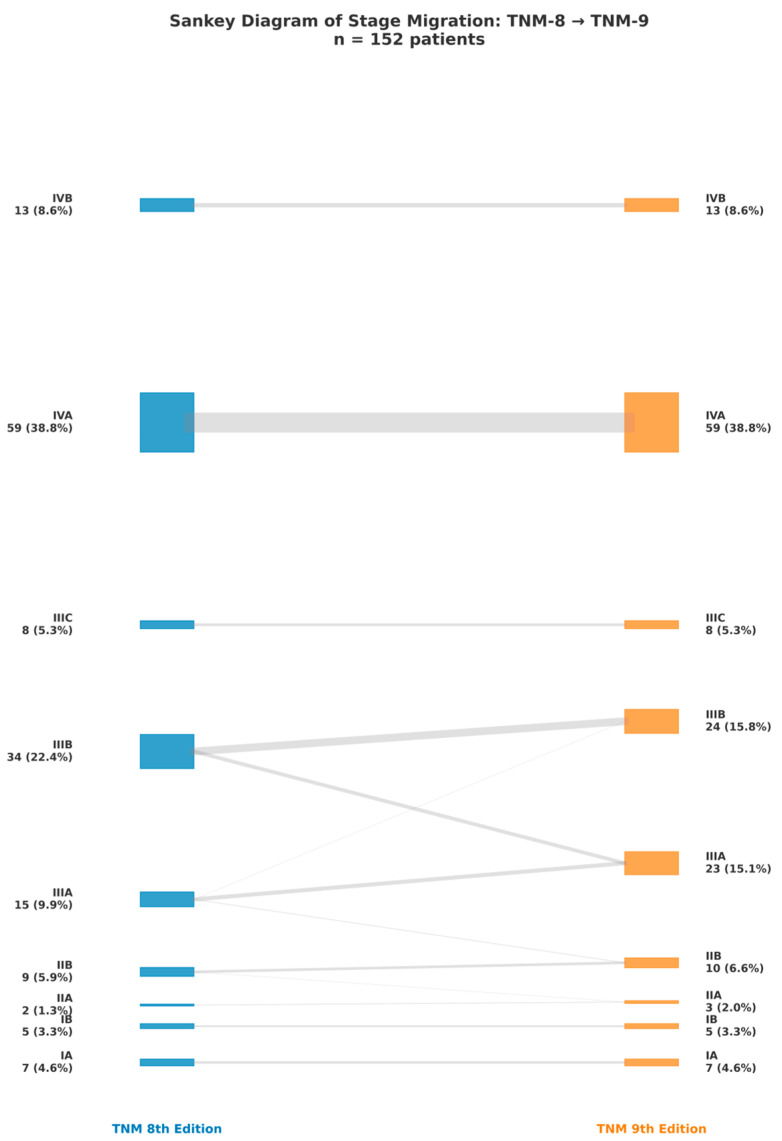
Sankey diagram illustrating patient migration between TNM stages according to the 8th and 9th editions. Stages from the 8th edition are shown on the left, and stages from the 9th edition are shown on the right. The thickness of the gray flows reflects the number of patients transitioning from one stage to another, while the colored boxes indicate the proportion of each stage relative to the entire cohort (n = 152 patients). Blue: TNM 8; Orange: TNM 9.

The distribution of patients across TNM stages according to the 8th and 9th editions is illustrated in Figure 14. A redistribution of cases between stages can be observed, with a noticeable migration of patients to lower or higher categories depending on the reclassification criteria. The proportions of each stage before and after reclassification are indicated next to the corresponding labels, showing both the absolute number of patients and their percentage within the cohort. The visual comparison reveals small yet clinically significant changes, notably an increase in the percentage of patients categorized as stage IIIA in the 9th edition.

### 3.4. Distribution of Histological Subtypes Across TNM 9 Stages

**Figure 15:**
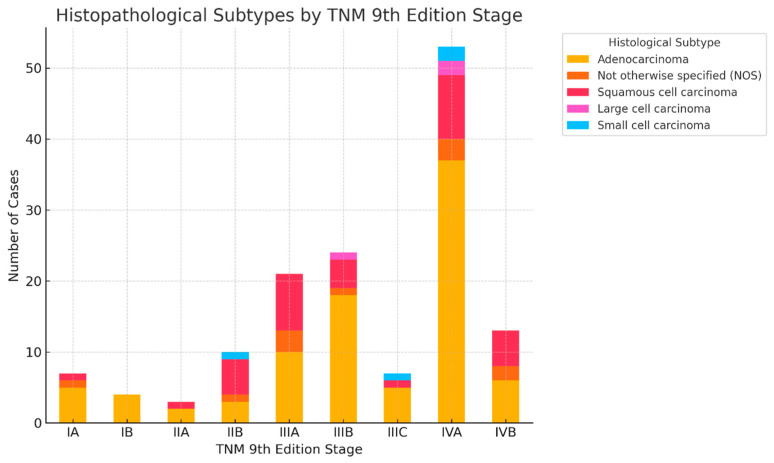
Distribution of histopathological lung cancer subtypes across TNM 9th Edition stages.

As shown in Figure 15, adenocarcinoma predominated across all stages, with the highest representation in stages IIIA and IIIB. Squamous cell carcinoma was mainly observed in stages IIB–IIIA, while small cell and large cell carcinomas clustered in more advanced stages. These findings highlight the histology-dependent distribution of reclassified cases in TNM 9.

### 3.5. Staging Transitions by Histology

**Figure 16:**
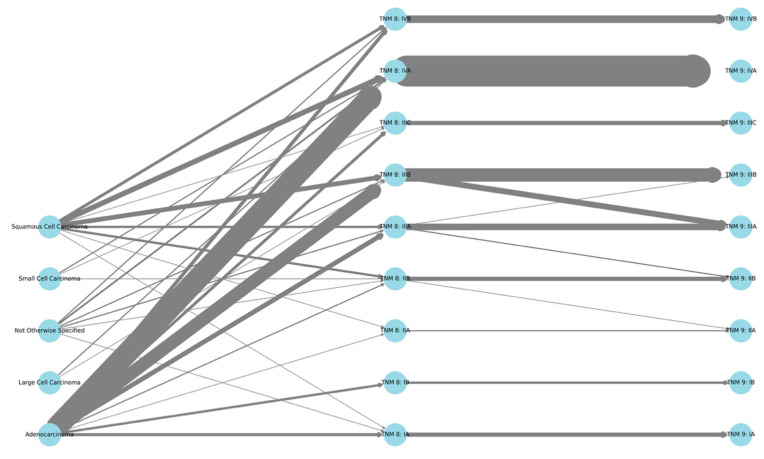
Alluvial diagram showing the staging transitions of lung cancer patients with defined histological subtypes from TNM 8 to TNM 9. The lines represent the migration of individual cases between stages, with thicker lines indicating a higher number of patients.

This diagram (Figure 16) illustrates the progression of patients diagnosed with the main histological subtypes of lung cancer: adenocarcinoma, squamous cell carcinoma, small cell carcinoma, large cell carcinoma, and NOS (not otherwise specified), as categorized by the 8th and 9th editions of the TNM staging system. Each node represents a staging category, and the width of the connections between them indicates the number of patients who transitioned from one stage to another. Only patients with a definitely documented histological subtype were incorporated into the analysis. This visualization highlights staging concordance and reclassification trends between the two TNM editions, elucidating the clinical implications of the revised staging guidelines.

### 3.6. Subtype-Specific Staging Comparisons

**Figure 17:**
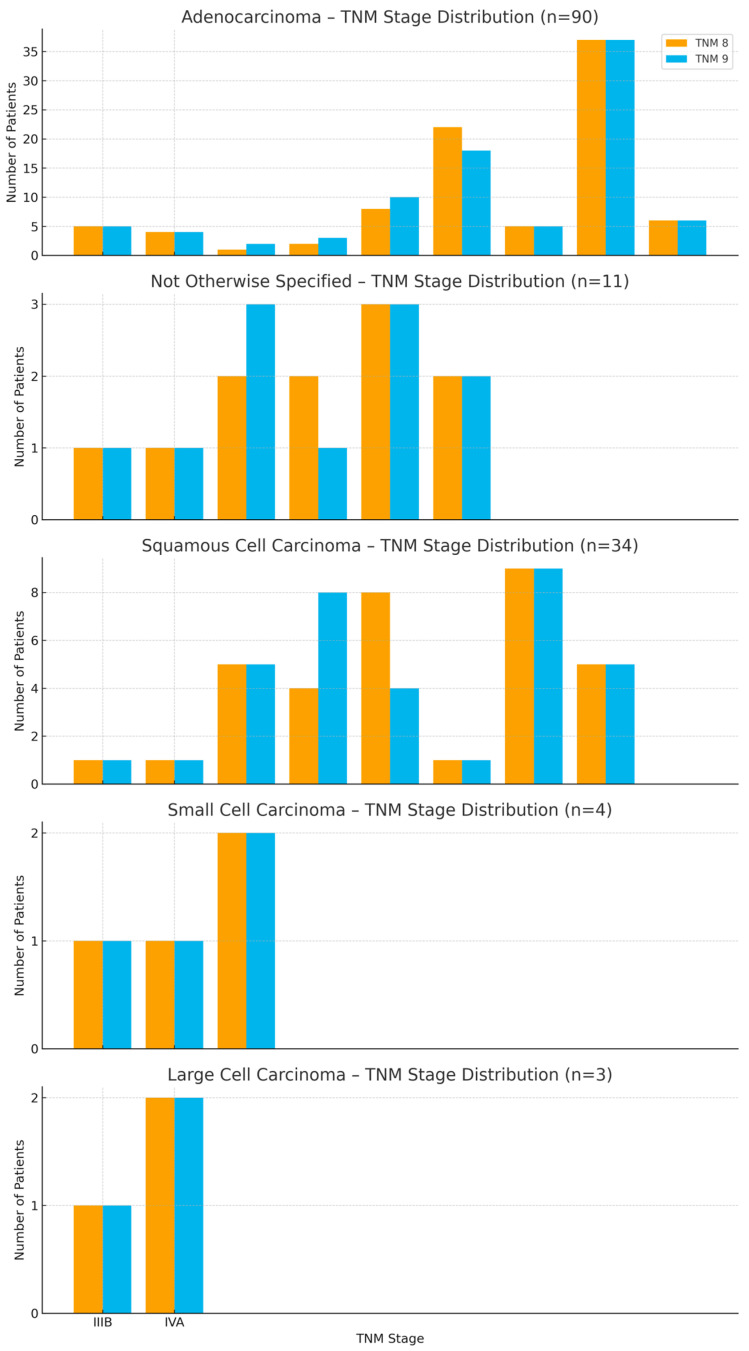
Comparison of TNM 8 and TNM 9 staging distributions across major lung cancer histological subtypes.

Figure 17 compares TNM 8 and TNM 9 distributions by histological subtype, showing both upstaging and downstaging. Reclassification was more frequent in squamous cell carcinoma and small cell carcinoma, underlining that TNM 9 captures biological and anatomical distinctions across subtypes.

### 3.7. Stage Variability Across Histologies

**Figure 18:**
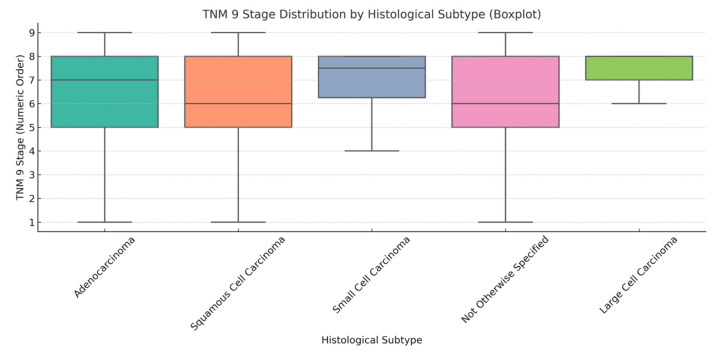
Boxplot of TNM 9 stage distribution by lung cancer histological subtype.

This boxplot (Figure 18) illustrates the distribution of TNM 9 stages among the principal histological subtypes of lung cancer. Stages have been changed into an ordinal number scale (IA = 1 to IVB = 9) to facilitate visual comparison of central tendency and variability. Each box illustrates the interquartile range (IQR) of stages for a certain histology, with medians highlighted and outliers represented as distinct dots. Adenocarcinoma exhibits the greatest stage distribution, encompassing both early and advanced stages, whereas small cell and large cell carcinomas predominantly concentrate in more advanced stages. This visualization illustrates the variability in disease presentation among histological subtypes and may indicate disparities in tumor biology, detection time, and clinical progression.

## 4. Discussion

### 4.1. Interpretation of Staging Shifts

The comparative analysis of the 8th and 9th editions of the TNM classification demonstrated both upward and downward reclassifications, with a noticeable predominance of downstaging. McNemar’s test confirmed that the adjustments were statistically significant, demonstrating that the 9th edition criteria exhibit greater sensitivity in detecting disease features associated with advanced stages [15].

A significant change was noted in stage IIIA, which demonstrated a substantial increase in patient numbers, suggestive of reallocation from stages IIB and IIIB. The modifications are mostly attributed to the division of the N2 category into N2a and N2b, along with the division of M1c into M1c1 and M1c2. The new categorization improves anatomical precision by differentiating between single and multiple nodal stations or metastatic sites, therefore influencing prognostic categorization. These modifications were suggested and confirmed using the IASLC database and are highlighted in radiology-oriented summaries [10].

External series have already documented similar redistribution. A Brazilian single-center re-staging of TNM 8 cases using TNM 9 criteria identified 34 patients who were downstaged—predominantly from IIIB to IIIA due to N2a classification—and 8 patients who were upstaged from IIIA to IIIB. Overall survival trends favored the downstaged cohort, though without statistical significance, thereby supporting the clinical justification for our transition to IIIA [16].

Similarly, a 2025 cohort concentrating on systematic mediastinal staging indicated that N2b is associated with significantly greater mortality than N2a, therefore strengthening the prognostic explanation for the N2 subdivision observed in our findings [17].

The reclassification of distant metastasis (M1c → M1c1 for a single extrathoracic organ versus M1c2 for multiple organs) did not modify the overall stage IV classification (both remain IVB); nevertheless, it improves phenotypic particularity for clinical trials and prognostic assessments consistent with our observation that the total volume of stage IV remained unchanged despite the re-labeling. In summary, our reclassification movements, especially the growth of IIIA, align with international standards and the IASLC rationale for TNM 9: improved nodal/metastatic stratification that enhances prognostic differentiation and better correlates stage groups with modern therapeutic strategies [10].

### 4.2. Impact on Treatment Eligibility and Prognosis

The transition from TNM 8 to TNM 9 staging directly affects patient eligibility for specific therapy options. The revised classifications of nodal involvement (N2a vs. N2b) and metastatic burden (M1c1 vs. M1c2) enable clinicians to more precisely categorize patients into surgical, multimodal, or palliative treatment options.

Our results align with Hwangbo B. et al.’s study [18], which confirmed the prognostic significance of N2 subdivision. In that multicenter investigation, N2b disease was correlated with significantly poorer overall survival compared to N2a, even after adjustment for other clinicopathological factors. N2b exhibited a higher hazard ratio for mortality, highlighting the necessity of distinguishing between single and numerous mediastinal nodal stations. This stratification improves risk assessment and facilitates adjusting therapy strategies to specific patient profiles.

According to the National Cancer Institute PDQ NSCLC guidelines [12], patients with stage IIIA disease, especially those with single-station N2 involvement, may qualify for induction chemotherapy followed by surgery. On the other hand, stage IIIB or IIIC disease typically needs definitive concurrent chemoradiation, as surgical intervention is rarely viable. Advancing to these higher categories, as observed in our study, can therefore change the therapeutic intent from potentially curative multimodal strategies to definitive nonsurgical treatment.

Our findings, specifically the noted net downstaging indicate that TNM 9 enhances the correlation between anatomical extent and prognosis, therefore reducing variation between stage groups. This improved granularity assists in determining suitable treatment intensity, enables more precise guidance for patients about anticipated outcomes, and improves the comparability of clinical trial cohorts.

Overall, TNM 9 reclassification does not simply reorganize categories; it transforms therapeutic decision-making and prognostic evaluation, guaranteeing that staging more accurately represents the reality of tumor biology and disease dissemination.

In our institution, these staging refinements have already influenced treatment decisions. Specifically, the downstaging of selected IIIA cases (e.g., T3N2a reclassified from IIIB to IIIA) has expanded eligibility for multimodal strategies including induction therapy followed by surgery. Conversely, the upstaging of patients with N2b disease has led to prioritization of definitive chemoradiation protocols rather than surgical intervention. These real-world adjustments illustrate the practical clinical relevance and ease of adoption of TNM 9 in daily multidisciplinary decision-making.

### 4.3. Comparison with International Data and Literature

Our observations on stage distribution between TNM 8 and TNM 9 correspond with trends identified in worldwide cohorts, although with significant histology- and stage-specific distinctions. The findings of our study align with those reported by Asamura et al. in the IASLC Lung Cancer Staging Project, which analyzed an extensive international dataset to inform the revisions for the 9th edition of the TNM classification. Similar to their results, our data demonstrate a measurable redistribution of cases across stages, predominantly characterized by downstaging, reflecting the impact of the updated staging criteria. Both studies utilized a stage-by-stage comparison between TNM 8 and TNM 9, highlighting categories most affected by reclassification. However, our cohort differs substantially in scope and clinical context: while the IASLC dataset incorporated multi-institutional, multi-national data covering a wide spectrum of clinical settings, our analysis focused on a single-center cohort, predominantly patients undergoing CT-guided transthoracic lung biopsy, providing a histology-specific perspective on stage migration. This narrower but more histologically detailed approach offers complementary insights into how TNM 9 impacts patient classification in a real-world diagnostic setting, particularly within a Central-Eastern European population, which remains underrepresented in global staging datasets [7].

Histologically, our data confirm the preponderance of adenocarcinoma throughout all phases, particularly in advanced stages (IIIA–IVB). This pattern corresponds to global registries, including GLOBOCAN-associated epidemiological studies [1,2], which explain the elevated prevalence of adenocarcinoma due to shifts in smoking behaviors, increasing incidence among never-smokers, and the biological tendency for early distant metastasis. Conversely, squamous cell carcinoma is predominantly restricted to locally advanced stages (IIB–IIIA), as documented in Brazilian [16] and Turkish [17] studies.

Comparisons specific to stages further corroborate the prognostic significance of histology-stratified staging. In our dataset, adenocarcinoma patients were more often downstaged from IIIB to IIIA or from IIIA to IIB according to TNM 9, indicating the sensitivity of the N2a/N2b modification in identifying clinically significant nodal disease. In contrast, squamous cell carcinoma cases demonstrated equitable reclassification trends, indicating that tumor size and local invasion are more significant factors in their staging determination. Demirdöğen et al. discovered that individuals with N2b disease exhibited a markedly elevated two-year mortality risk (HR = 2.78), but those with N2a disease did not demonstrate a similar prognostic disadvantage. These findings underscore the therapeutic importance of categorizing the N2 in the updated classification.

In evaluating the extent of stage shifts, our IIIA increase (+53.3% relative change) exceeds the +37% reported in the Brazilian single-center reclassification study [16], possibly due to variations in referral patterns, imaging technology, and criteria for surgical eligibility evaluation. Nonetheless, the percentage of downstaged IIIA patients (e.g., T3N2a transitioning from IIIB) stays consistent, underscoring the worldwide trend that TNM 9 enhances surgical eligibility in specific situations.

Furthermore, preliminary evaluations by radiology specialists validate the minor changes to the T descriptor in TNM 9, while emphasizing the predictive significance of the recently included histopathologic markers: STAS (spread through air spaces) and features like vascular or perineural invasion have been advocated as auxiliary descriptors to improve TNM precision [19].

Our findings, along with external data, indicate that TNM 9 improves anatomical precision, especially for adenocarcinoma with complex nodal involvement, while maintaining prognostic differentiation across histologies. This advanced staging facilitates deeper multidisciplinary planning and may enhance survival results by correlating therapy intensity with biological and anatomical disease attributes.

A limitation of our analysis is that long-term survival data were not available, as all patients included in this study were diagnosed in 2025 and follow-up is still ongoing. Therefore, we were unable to directly verify whether TNM 9 stage groupings are more consistent with long-term outcomes compared to TNM 8. Future research at our institution will address this limitation by analyzing earlier cohorts of lung cancer patients (diagnosed 5–10 years ago), for which sufficient follow-up exists to perform outcome analyses, including Cox regression models.

## 5. Conclusions

The implementation of the 9th edition of the TNM classification for lung cancer has resulted in a notable reallocation of stages, characterized by a significant tendency towards downstaging, as confirmed by statistical analysis. One of the most notable findings in the transition from TNM 8 to TNM 9 is the redefinition of stage IIIA disease. This stage, previously characterized by marked heterogeneity, encompassed a wide range of tumor sizes and nodal involvement that often translated into disparate prognoses within the same category. The 9th edition addressed these inconsistencies by refining both tumor and nodal descriptors and redistributing cases to neighboring stages when appropriate. As a result, stage IIIA now represents a more homogeneous group, with improved prognostic stratification and clearer therapeutic implications. These changes are particularly relevant for surgical decision-making and multimodal treatment approaches, underscoring the importance of the updated classification in guiding contemporary clinical practice.

Histological analysis indicated a predominance of adenocarcinoma in advanced stages and an increased probability of reclassification into stages IIIA/IIIB, aligning with international data that demonstrate a strong association between adenocarcinoma and occult multi-station nodal involvement.

The findings confirm the therapeutic significance of the newly established subcategories, directly influencing eligibility for surgical and multimodal treatments while enhancing prognostic consistency within stage groups.

The implementation of TNM 9 indicates not only a category reorganization but also an advancement in staging that more precisely mirrors tumor biology and clinical reality therefore enhancing multidisciplinary treatment planning and improving worldwide comparability of patient cohorts.

## Figures and Tables

**Figure 1:**
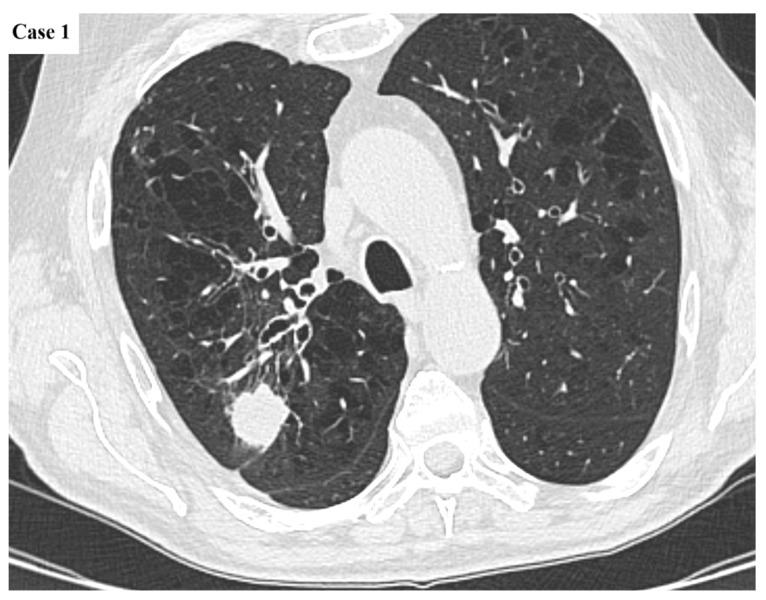
Axial CT image of a 26 mm lobulated mass located in the posterior segment of the right upper lobe. There is minimal tethering to the pleural surface. The large airways exhibit mild wall thickening and varicose bronchial dilatation, whereas the background lung parenchyma presents with diffuse centrilobular emphysematous changes.

**Figure 2:**
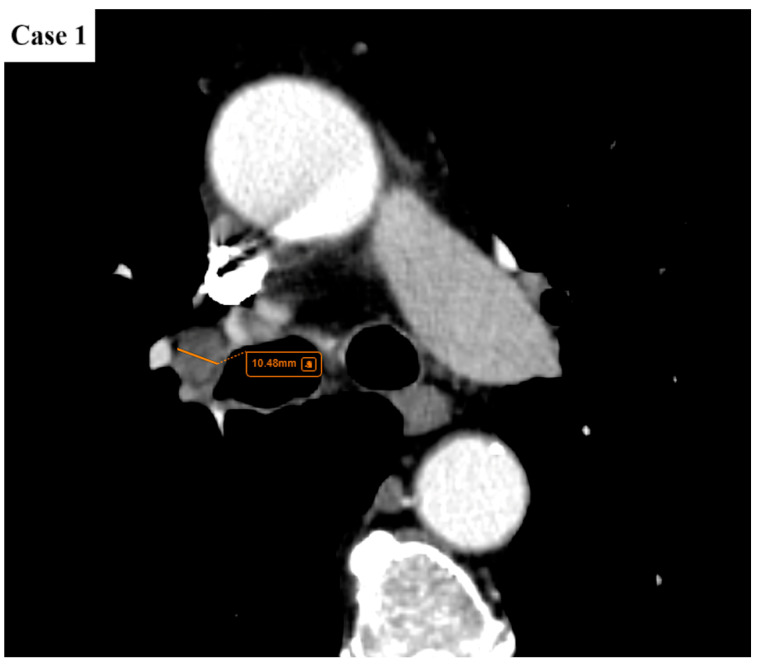
There is a single enlarged lymph node (short axis diameter/SAD of maximum 10.5 mm) in the right hilar region. No satellite intrapulmonary nodules. No pleural or pericardial effusions. The rest of the viscera appear unremarkable. The pulmonary mass, pathologically proven large cell neuroendocrine carcinoma, would be classified as T1cN1M0 (stage IIB according to TNM 8 and stage IIA according to TNM 9).

**Figure 3:**
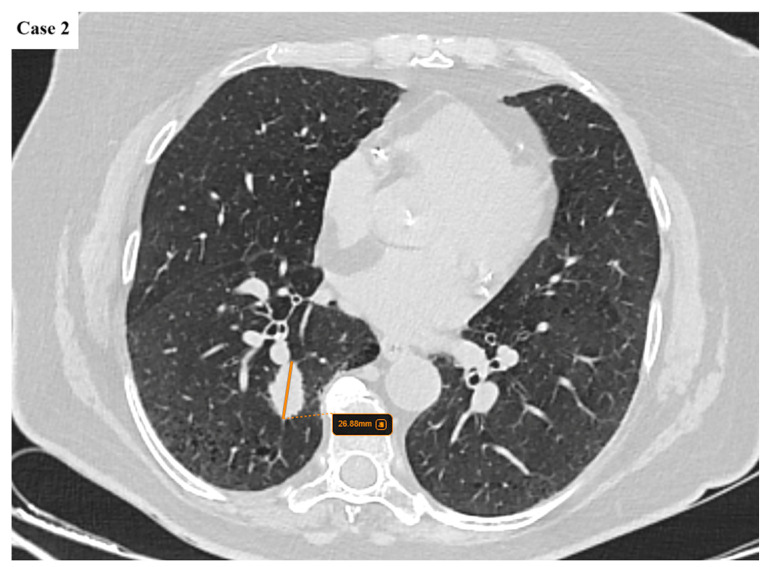
A 27 mm lobulated lung mass with spiculated margins is found in the posterior segment of the right lower lobe. Emphysematous changes are noted in the lower lobes.

**Figure 4:**
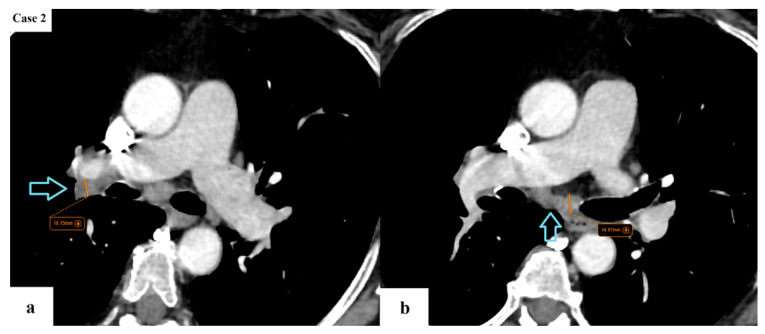
A few enlarged lymph nodes (blue arrows) with a SAD of maximum 11 mm are located in the ipsilateral hilar (**a**) and subcarinal (**b**) stations. The remainder of the viscera does not appear to present with any suspicious lesions. The lung tumor would be classified as T1cN2aM0 (stage IIIA according to TNM 8 and stage IIB according to TNM 9).

**Figure 5:**
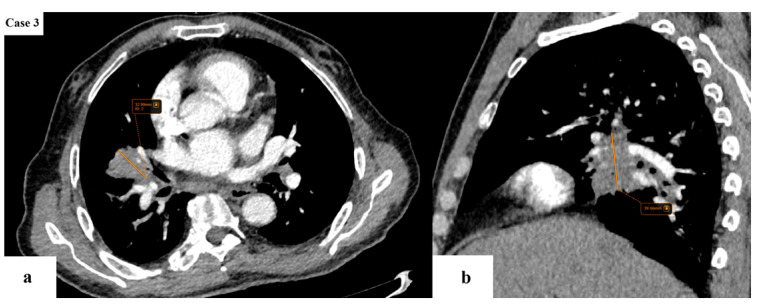
Axial (**a**) and sagittal (**b**) CT reconstructions of a 39 mm central lung mass located in the lateral segment of the right middle lobe, with extension into the superior segments of the right lower lobe.

**Figure 6:**
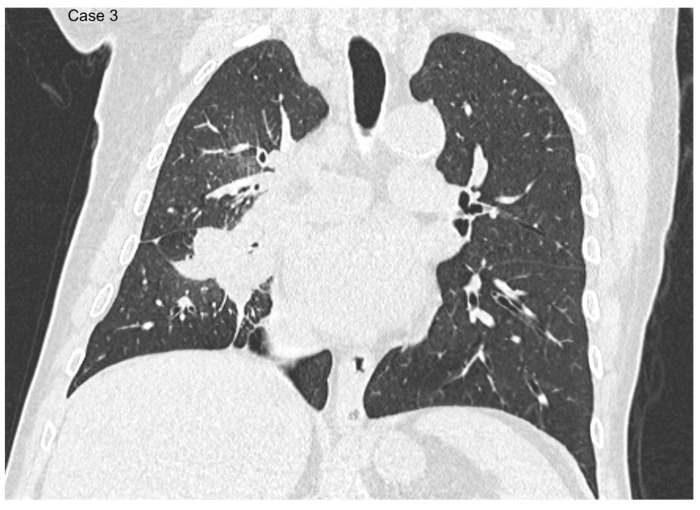
Coronal reformat shows the lesion traversing the right oblique fissure.

**Figure 7:**
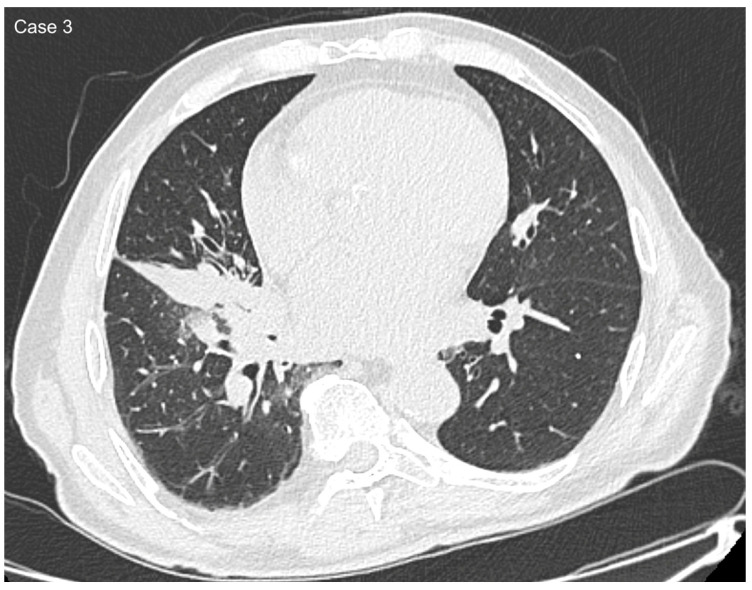
Axial CT image shows discrete ground-glass changes surrounding the lesion, as well as subsegmental collapse due to infiltration of the lateral bronchus of the middle lobe.

**Figure 8:**
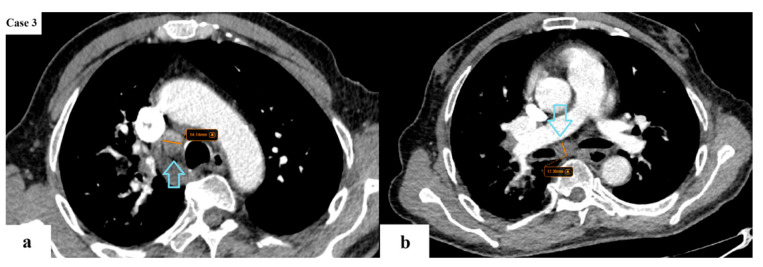
A few enlarged lymph nodes (blue arrows) (SAD of maximum 14 mm) are found in the ipsilateral mediastinal (**a**) and subcarinal (**b**) stations. No other intrapulmonary tumor nodules. No pleural or pericardial effusions. The rest of the viscera appear unremarkable. This is a case of a pathology-proven NSCLC that would be classified as T2aN2M0 (stage IIIA) according to TNM 8 and T2aN2bM0 (stage IIIB) according to TNM 9.

**Figure 9:**
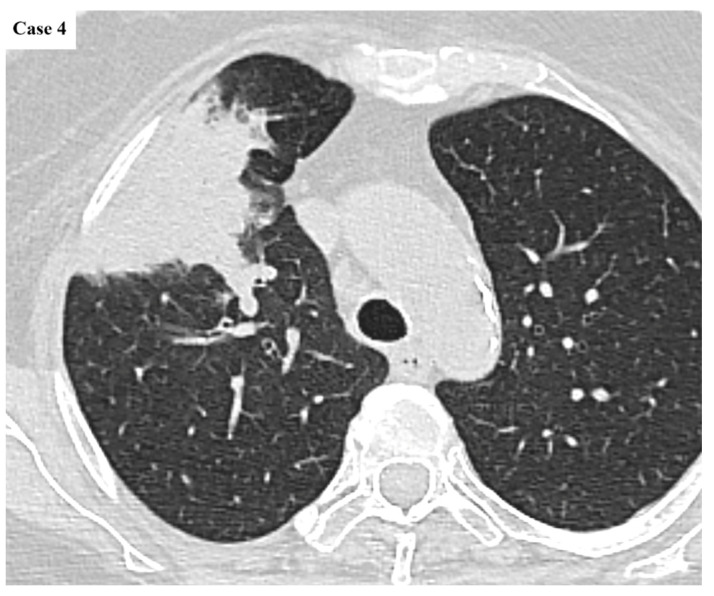
Axial CT image of a lung mass found peripherally in the anterior segment of the right lung.

**Figure 10:**
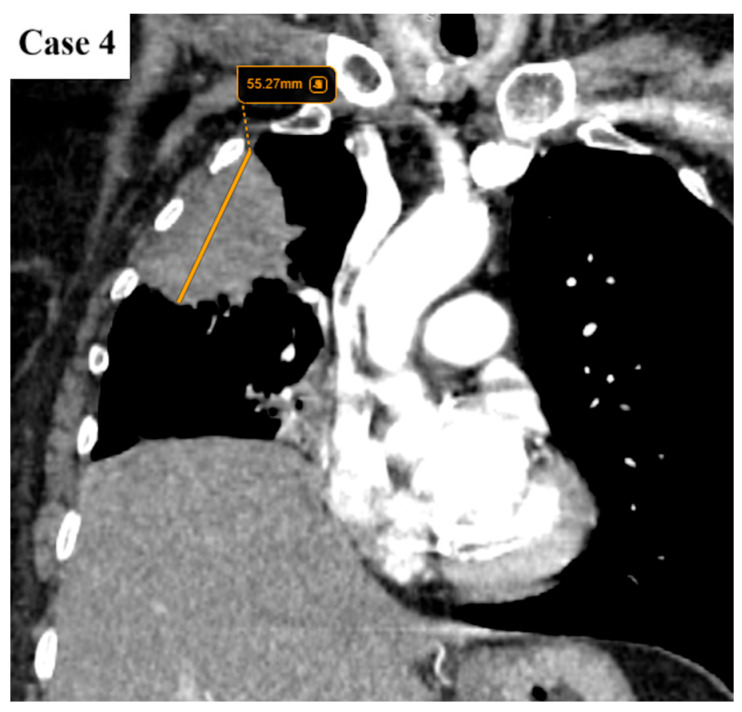
Coronal reformat indicates that the lesion has a maximum diameter of 55 mm. There are no signs of chest wall invasion. The right hemidiaphragm is elevated due to hepatomegaly.

**Table 1:** Baseline demographic, clinical, and imaging characteristics of the study population (n = 152).

Age (years)	Mean 66.4 ± 9.2 (range 40–87)
Sex	Male: 107 (70.4%) Female: 45 (29.6%)
Smoking status	Current: 71 (46.7%) Former: 49 (32.2%) Never: 32 (21.1%)
BMI (kg/m^2^)	Median 25.3 (IQR 22.3–28.5)
Pack-years	Median 30 (IQR 20–50)
Stage at diagnosis (TNM 8)	IA: 5 (3.3%) IB: 6 (3.9%) IIA: 7 (4.6%) IIB: 9 (5.9%) IIIA: 23 (15.1%) IIIB: 29 (19.1%) IIIC: 12 (7.9%) IVA: 46 (30.3%) IVB: 15 (9.9%)
Histology	Adenocarcinoma: 84 (55.3%) Squamous cell carcinoma: 39 (25.7%) Small cell: 12 (7.9%) Large cell: 7 (4.6%) Other/unspecified: 10 (6.6%)
Tumor size (cm)	Median 4.2 (IQR 2.6–5.9)
Tumor location	Upper lobe: 80 (52.6%) Lower lobe: 66 (43.4%) Diffuse involvement: 6 (3.9%)
Imaging features	Spiculated margins: 88% Central necrosis: 38.4% Cavity: 10.4% Lymphangitic spread: 43.2%

Note: Stage at diagnosis was determined according to the 8th edition of the TNM classification, which was still in use at our institution in early 2025. Cases were subsequently reclassified according to TNM 9 for comparative analyses.

## Data Availability

The information is contained within this article in its entirety. For additional information, please feel free to inquire with either the original author or the corresponding author. Public access to data is restricted as a result of the patient privacy standards that regulate the handling of clinical data.
